# Antimicrobial resistance profile and prevalence of extended-spectrum beta-lactamases (ESBL), AmpC beta-lactamases and colistin resistance (*mcr*) genes in *Escherichia coli* from swine between 1999 and 2018

**DOI:** 10.1186/s40813-020-00146-2

**Published:** 2020-04-02

**Authors:** Laia Aguirre, Anna Vidal, Chiara Seminati, Montse Tello, Noelia Redondo, Laila Darwich, Marga Martín

**Affiliations:** 1 Departament de Sanitat i d’Anatomia Animals, Universitat Autònoma de Barcelona, Edifici V, Travessera dels Turons, 08193 Bellaterra, Spain; 2 UAB, Centre de Recerca en Sanitat Animal (CReSA, IRTA-UAB), Campus de la Universitat Autònoma de Barcelona, 08193 Bellaterra, Spain

**Keywords:** Antimicrobial resistance, *Escherichia coli*, Pig, *Colistin-mcr genes*, ESBL

## Abstract

The frequent usage of antibiotics in livestock has led to the spread of resistant bacteria within animals and their products, with a global warning in public health and veterinarians to monitor such resistances. This study aimed to determine antibiotic resistance patterns and genes in pig farms from Spain during the last twenty years. Susceptibility to six antibiotics commonly used in pig production was tested by qualitative (disk diffusion) and quantitative (minimum inhibitory concentration, MIC) methods in 200 strains of *Escherichia coli* which had been isolated between 1999 and 2018 from clinical cases of diarrhoea in neonatal and post-weaned piglets. Results showed resistance around 100% for amoxicillin and tetracycline since 1999, and a progressive increase in ceftiofur resistance throughout the studied period. For colistin, it was detected a resistance peak (17.5% of the strains) in the 2011–2014 period. Concerning gentamicin, 11 of 30 strains with intermediate susceptibility by the disk diffusion method were resistant by MIC. Besides, the most frequent antimicrobial resistance genes were the extended-spectrum beta-lactamase (ESBL) *bla*_CTX-M_ (13.5% of strains, being CTX-M-14, CTX-M-1 and CTX-M-32 the most prevalent genomes, followed by CTX-M-27, CTX-M-9 and CTX-M-3), AmpC-type beta-lactamase (AmpC) *bla*_CMY-2_ (3%) and colistin resistance genes *mcr*-4 (13%), *mcr*-1 (7%) and in less proportion *mcr*-5 (3%). Interestingly, these *mcr* genes were already detected in strains isolated in 2000, more than a decade before their first description. However, poor concordance between the genotypic *mcr* profile and the phenotypical testing by MIC was found in this study. These results indicate that although being a current concern, resistance genes and therefore antimicrobial resistant phenotypes were already present in pig farms at the beginning of the century.

## Background

Levy et al. (1976) observed the direct link between the usage of antibiotics in livestock and the apparition of resistances in human pathogens by plasmid transfer [1]. Nowadays, antibiotics are still extensively used in the animal husbandry sector, especially in those farming activities with the most intensive systems such as pork production. Pig industry is the most important livestock sector in Spain. Recent studies show that the percentage of resistance to last-resort drugs such as colistin is considerably higher than in other European regions [2, 3]. Following these reports and considering the worldwide increasing prevalence of antimicrobial resistance (AMR) in food-producing animals, the aim of this study was to determine the present situation of AMR in pig farms from Spain and its evolution since 1999, as well as the prevalence of the most common resistance genes (colistin resistance plasmids (*mcr*), genes encoding for extended-spectrum beta-lactamases (ESBL), AmpC-type beta-lactamases (AmpC) and carbapenemases).

## Methods

One hundred and sixty pathogenic *E. coli* strains isolated from swine enteric clinical cases were randomly recovered from the strain collection of the Infectious Diseases Laboratory of the Veterinary Faculty of Barcelona (Spain) between 1999 and 2014 (8–16 strains per year). Those cases came from farms of different counties of Spain with neonatal or post-weaning diarrhoea problems. In addition, forty different *E. coli* strains isolated throughout 2017 and 2018 were collected from ten farms in Catalonia (NE of Spain). The primary objective was to determine the evolution of antimicrobial resistances in *E. coli* strains since 1999 against the most commonly used antibiotics in swine. A second objective was to study the presence of resistance genes (ESBL, carbapenemase and *mcr*) by molecular methods in all the selected strains.

For this purpose, strains from the collection were recovered in brain heart infusion and then streaked on blood agar and MacConkey agar, both incubated aerobically at 37 °C overnight. Faecal samples collected in 2017 and 2018 were plated in the same culture media and conditions in order to isolate *E. coli* strains. The qualitative disk diffusion method on Mueller Hinton agar was used to test susceptibility to amoxicillin, tetracycline, gentamicin, enrofloxacin, ceftiofur and colistin according to the Clinical and Laboratory Standards Institute (CLSI) [3, 4] using the antibiotic disks and breakpoints shown in Table 1. Amoxicillin-clavulanic resistance was tested in strains resistant to ceftiofur to predict the production of AmpC beta-lactamase. At the same time, the minimum inhibitory concentration (MIC) of colistin was determined for all strains by broth microdilution method in 96-wells microplates, using a dilution prepared from colistin sulphate of 21958 IU/mg (731,93 μg/mg) potency [4]. Additionally, for strains with resistant, intermediate and sensitive but close to the breakpoint results to gentamicin, MIC values were also determined by broth microdilution with a dilution from gentamicin sulphate. Both MIC microdilution procedures were manually prepared and performed according to CLSI standards, using the reference strain *E. coli* ATCC 25922 as control in each plate. CLSI breakpoints shown in Table 2 were used.

**Table 1:** Concentrations and breakpoints of the antibiotic disks used for the disk diffusion method

Antibiotic^a^	Concentration (μg/mg)	Breakpoint (mm)		Reference^b^
		S	R	
Amoxicillin	25	≥ 17	≤ 13	CLSI M100; human [5]
Amoxicillin - clavulanic	30	≥ 18	≤ 13	CLSI M100; human [5]
Ceftiofur	30	≥ 21	≤ 17	CLSI VET08; cattle *E. coli* and swine *Salmonella cholerasuis* [6]
Colistin	50	≥ 15	< 15	CA-SFM; veterinary [7]
Enrofloxacin	5	≥ 23	≤ 16	CLSI VET08; dog, cat and poultry [6]
Gentamicin	10	≥ 16	≤ 12	CLSI VET08; dog, horse [6]
Tetracycline	30	≥ 15	≤ 11	CLSI M100; human [5]

^a^Antibiotic disks: BBL™ brand. ^**b**^CLSI veterinary breakpoints were preferably used. If not available, CLSI human or CA-SFM veterinary breakpoints were used

**Table 2:** Potency and breakpoints used for the microdilution methods for colistin and gentamicin

Antibiotic^a^	Potency (μg/mg)	Breakpoint (μg/mL)		Reference^b^
		S	R	
Colistin	731.93	≤ 2	> 2	CLSI M100; human [5]
Gentamicin	614	≤ 2	≥ 8	CLSI VET08; dog, horse [6]

^a^Antibiotic powder: Sigma®. ^b^CLSI veterinary breakpoints were preferably used. If not available, CLSI human breakpoints were used

The most common genes coding for ESBL (*bla*_CTX-M_, *bla*_TEM_, *bla*_SHV_), AmpCs (*bla*_CMY-1_, *bla*_CMY-2_), carbapenemases (*bla*_OXA_) and colistin resistance (*mcr*-1, *mcr*-2, *mcr*-3, *mcr*-4 and *mcr*-5) genes were analysed by PCR in all strains as previously described [8]. Controls for all genes were provided by Dr. Migura (IRTA-CReSA, Bellaterra, Spain). Amplified PCR products were Sanger sequenced for verification at the Genomic and Bioinformatics Service of the Universitat Autònoma de Barcelona (Bellaterra, Spain). Sequences were analysed by using BioEdit software and blasted against the public database (National Center for Biotechnology Information, NCBI).

Statistics were applied for determining antimicrobial resistance differences among the periods studied using the Chi-Square Test (Fisher Exact *P*-values) and graphed using the program Excel. Antimicrobials with statistical differences were further analysed by T-Square. Finally, associations between *mcr* genes and MIC values were assessed by Odds-ratio tests.

## Results

**Fig. 1:**
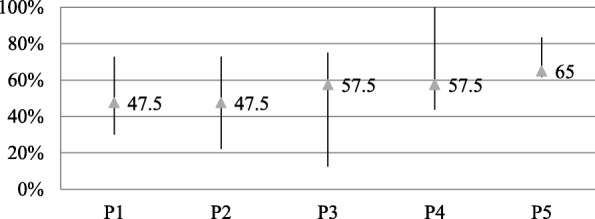
Minimum, maximum and mean percentage of MDR during the studied years, grouped in five periods (P1 = 1999–2002, P2 = 2003–2006, P3 = 2007–2010, P4 = 2011–2014, P5 = 2017 and 2018) according to the results obtained by disk diffusion with the exception of colistin, for which the results of the MIC were considered. Strains with intermediate susceptibility to gentamicin by disk diffusion but resistant MIC were considered resistant. The line represents the interval of values in the defined period from the minimum to the maximum percentage, and the mean (number of MDR strains in the period divided by the total of strains of the period) is represented by the triangle

The disk diffusion tests demonstrated that 110 of 200 strains (55%) were multidrug-resistant (MDR), namely resistant to three or more antibiotics. Because of the low number of samples per year, strains were grouped in 4-year periods from 1999 to 2014 (P1 = 1999, 2000, 2001 and 2002, P2 = 2003, 2004, 2005 and 2006, P3 = 2007, 2008, 2009 and 2010, P4 = 2011, 2012, 2013 and 2014) with 40 strains per period, and a last group of strains from 2017 and 2018 (P5), of 40 samples. As shown in Fig. 1, no significant changes in the MDR percentages were observed over time (*P* > 0.05). However, the percentage of MDR was significantly higher in P5 (65%) than in P1 and P2 (47.5%) (*P* < 0.02).

**Fig. 2:**
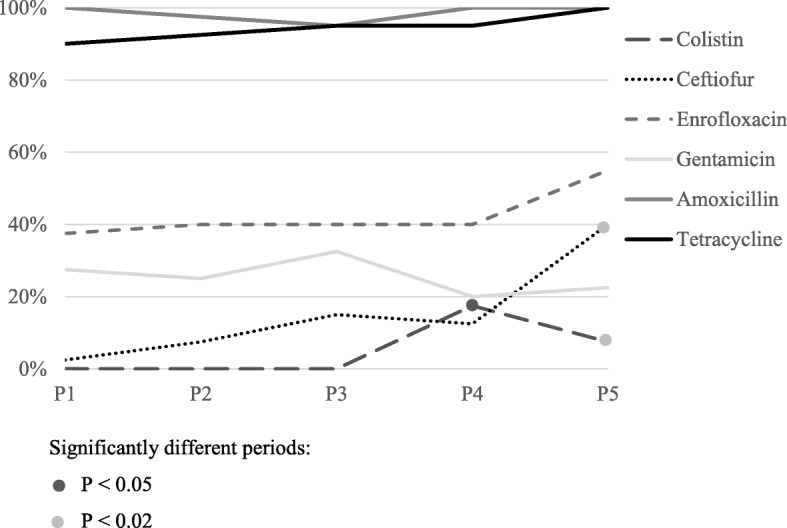
Percentages of resistance to each of the 6 antibiotics in the five defined periods (P1 = 1999–2002, P2 = 2003–2006, P3 = 2007–2010, P4 = 2011–2014, P5 = 2017 and 2018) according to the results obtained by disk diffusion with the exception of colistin, for which the results of the MIC were considered. Strains with intermediate susceptibility to gentamicin by disk diffusion but resistant MIC were considered resistant

Amoxicillin resistance was observed in 98.5% (197/200) of the strains. There were no significant differences in amoxicillin resistance among studied periods (*P* > 0.05), as can be seen in Fig. 2. Similarly, 94.5% (189/200) strains were resistant to tetracycline, with no significant changes between 1999 and 2018 (*P* > 0.05). Resistance to ceftiofur was detected in 15.5% of the samples (31/200). As can be observed in Fig. 2, resistance to this antibiotic was significantly higher in P5 than in the earlier periods (*P* < 0.02). Enrofloxacin showed a level of resistance of 42.5% (85/200), a percentage that has remained practically stable in these past twenty years (*P* > 0.05). Gentamicin resistance was detected in 25.5% (51/200) strains, with no significant differences (*P* > 0.05) over the studied years. Thirty strains (15%) presented intermediate susceptibility to gentamicin. Among these, 36.67% (11/30) had a resistant MIC when tested by microdilution. Of the eight strains with a sensitive result to gentamicin by disk diffusion but close to the breakpoint (16 mm), five presented resistant MIC values. The qualitative results of resistance to colistin did not show significant changes throughout the period (*P* > 0.05), with only 4.67% of resistant strains. In contrast, the MIC procedure demonstrated significant differences among the studied years (*P* < 0.02). Whereas there was no colistin resistance in neither of the three first periods, an increase was observed in P4 (*P* < 0.05), with 17.5% (7/40) of strains resistant to colistin. However, the percentage in 2017 and 2018 declined significantly (*P* < 0.02) to 7.5% (3/40) (Fig. 2).

The frequencies of the AMR genes are shown in Table 3. Only the ESBL gene *bla*_CTX-M_, was detected, in 13.5% (27/200) strains, which showed a slight a tendency to increase throughout the studied period (*P* < 0.05). Out of the 27 strains, the sequences of 18 strains could be obtained (Table 3). The most prevalent was *bla*_CTX-M-14_, with 33.33% (6/18), followed by *bla*_CTX-M-1_ with 27.78%, *bla*_CTX-M-32_ with 16.67% (3/18) and *bla*_CTX-M-3_ and *bla*_CTX-M-9_ both in 5.56% (1/18) of the strains. No significant trend patterns were described for any of the other AMR genes. *bla*_CMY-2_ was detected in 3% (6/200) of strains. The *mcr*-4 was the most frequent colistin resistance gene, in 13% (26/200) strains. Both *mcr*-1 and *mcr*-5 were detected in 7% (14/200) and 3% (6/200) strains respectively. Out of the 43 strains positive to at least one *mcr* gene, forty (93.02%) had a sensitive MIC value (0.5–2 μg/mL), and seven (4.46%) of the 157 strains negative to *mcr* genes presented a resistant MIC. Therefore, no significant association was found between *mcr*-positivity and MIC results (*P* > 0.05). The MIC distribution by *mcr* PCR results can be observed in Table 4. *bla*_TEM_, *bla*_SHV_, *bla*_CMY-1_, *bla*_OXA_, *mcr*-2 and *mcr*-3 genes were not detected in any of the studied strains.

**Table 3:** Number of *E. coli* isolates positive to the different AMR genes and *bla*_CTX-M_ genotype sequencing results by year of isolation

Year (strains per year)	*mcr*-1	*mcr*-4	*mcr*-5	*bla*_CMY-2_	*bla*_CTX-M_	*bla*_CTX-M_ genotype
2000 (10)			2			
2001 (11)		2	3	2	1	
2002 (9)		1	1	2		
2003 (10)		5			1	
2004 (9)		3			1	
2005 (11)	3	2			2	CTX-M-14 (1) CTX-M-32 (1)
2006 (10)		1				
2007 (10)					2	CTX-M-14 (1)
2008 (10)	2	3				
2009 (8)	1			1	2	CTX-M-1 (1) CTX-M-14 (1)
2010 (12)		1			4	CTX-M-9 (1) CTX-M-32 (1)
2011 (16)	2	2		1	2	CTX-M-1 (2)
2012 (13)	2				2	
2013 (6)					1	
2014 (5)		3				
2017 (34)	3	1			9	CTX-M-1 (2) CTX-M-14 (3) CTX-M-32 (1) CTX-M-3 (1) CTX-M-27 (2)
2018 (6)	1	2				
**Total (%)**	14 (7)	26 (13)	6 (3)	6 (3)	27 (13.5)

**Table 4:** Number of strains by MIC colistin values and *mcr* PCR results

Colistin	MIC (μg/mL)	*mcr-*1	*mcr-*4	*mcr-*5	*mcr* absence^a^
Resistant	> 8	1	2	0	4
	4	0	0	0	3
Sensitive	2	2	2	0	44
	< 2	11	20	6	83

^a^*mcr-*1*, mcr-*2*, mcr-*3 *mcr-*4 and *mcr-*5 were tested

## Discussion

In this study, MDR *E. coli* strains were found in a high proportion of porcine diarrheic cases from Spanish farms since 1999. In addition, an increase of these MDR frequencies was observed in 2017 and 2018, being amoxicillin and tetracycline the antimicrobials with the highest levels of resistance (98.5 and 94.5% respectively). Similar resistance levels to these antibiotics have been previously described in enterobacteria from pig farms of Catalonia in 2002 [9, 10]. According to the EMA [11], tetracycline was the most sold antibiotic family for animal production in Spain from 2010 to 2015 and is still highly used in pigs and other species. Similarly, penicillins proved to be the second most consumed antibiotic family in the same report, which would explain the high levels of resistance to amoxicillin in the studied strains.

By contrast, resistance to ceftiofur was considerably lower (15.5%), as third and fourth generation cephalosporins are less used in livestock [11]. However, the ESBL increase described during the last decades [12] would account for the significant difference (*P* < 0.02) in ceftiofur resistance between P5 and the earlier periods.

On the other hand, enrofloxacin presented a level of resistance around 40% that has remained practically stable in these past twenty years. Although this fluoroquinolone is exclusively for veterinary use, previous studies have demonstrated that enrofloxacin treatments in pigs increases *Campylobacter coli* resistance to other fluoroquinolones such as ciprofloxacin [13], broadly used in humans. Out of the 28 strains resistant to ceftiofur, 16 (56.14%) presented resistance to amoxicillin-clavulanic, which has been described as an indicator of the presence of AmpC, although not highly specific [14].

Resistance to gentamicin in swine *E*. *coli* has been less reported than to other aminoglycosides like streptomycin [9, 15]. In this study, gentamicin resistance was detected in 25.5% (51/200) strains, with no significant differences (*P* > 0.05) over the studied years. The low but considerable percentage of intermediate strains (15%) and the fact that 36.67% (11/30) of these presented a resistant MIC suggests that disk diffusion results for testing gentamicin susceptibility should be interpreted with caution.

Colistin results from the qualitative MIC method were considered for the analysis, as the disk diffusion method has been described as not reliable when testing polymyxins susceptibility [5], and the results in the present study reinforce this fact. From 2013 to 2015, Spain was the European country with the highest sales of polymyxins for food-producing animals [11], which might explain the significant increase in colistin resistance in P4. The following significant decrease in colistin resistance in P5 might be related to the 97% reduction in the use of colistin in swine from 2015 to 2018 in Spain reported by the AEMPS [16].

In accordance with previous studies in humans and pigs [17], *bla*_CTX-M_ was the most frequent ESBL gene in our study, with upward trend throughout the years. On the other hand, *bla*_CMY-2_ and the six *bla*_CTX-M_ genotypes detected in this study (CTX-M-1, CTX-M-3, CTX-M-9, CTX-M-14, CTX-M-27 and CTX-M-32) had been already been reported in *E. coli* isolates from pigs [12, 18].

Since their first report by Liu et al. [19], *mcr* genes in *E. coli* strains have been worldwide described in both humans and livestock. It has been speculated that colistin use in animal production could be the original source of these genes [20]. Although *mcr*-4 was firstly described in 2017 by Carattoli et al. [21], in the present study *mcr*-4 (13%,) was already detected in *E. coli* strains isolated in 2001. Also, *mcr*-1 (7%,) was the second most prevalent colistin resistance gene, with the first positive strains detected in 2005. Finally, *mcr*-5, which was firstly described in 2017 by Borowiak et al. [22], was identified in four strains from 2001 and 2002, but it was not detected afterwards. The existence of strains positive to a *mcr* gene but with a sensitive MIC value suggests that, although they do not express it, strains with a “sensitive” phenotype could contain a *mcr* gene and might end up expressing it. Conversely, the seven strains found phenotypically resistant to colistin and negative for the *mcr* genes analysed, might suggest the presence of other *mcr* genes such as *mcr*-6 and *mcr*-8, originally described in pigs [23, 24] or other genes such as *pmrA* that can confer intrinsic resistance to colistin in *E*. *coli* [25] and have not been included in the study. Besides, colistin resistance could occur via chromosomal mutations, although this mechanism is often unstable [19].

## Conclusions

*E*. *coli* strains containing *bla*_CTX-M_ and *mcr*-4 and *mcr-*1 genes have been the most frequently detected in pig farms, since at least 20 years ago in the case of *mcr*-4. However, the poor concordance between the genotypic *mcr* profile and the MIC phenotypical testing observed in this study deserves further investigation. Finally, it is concerning the high levels of AMR for tetracycline and amoxicillin and the increase of cephalosporin and colistin resistance during these two decades. Thus, antimicrobial surveillance should be maintained, and research strengthened in new approaches to control of bacterial infections in pigs by reducing antibiotic consumption.

## Data Availability

The datasets generated during the current study are available from the corresponding author on reasonable request.

## Abbreviations

AMR: Antimicrobial resistance
ESBL: Extended-spectrum beta-lactamases
MDR: Multidrug-resistant
MIC: Minimal inhibitory concentration
